# Association between PSA values and surveillance quality after prostate cancer surgery

**DOI:** 10.1002/cam4.2663

**Published:** 2019-11-05

**Authors:** Christina Hunter Chapman, Megan E. V. Caram, Archana Radhakrishnan, Alexander Tsodikov, Curtiland Deville, Jennifer Burns, Alexander Zaslavsky, Michael Chang, John T. Leppert, Timothy Hofer, Anne E. Sales, Sarah T. Hawley, Brent K. Hollenbeck, Ted A. Skolarus

**Affiliations:** ^1^ Center for Clinical Management Research Veterans Affairs Ann Arbor Healthcare System Ann Arbor MI USA; ^2^ Department of Radiation Oncology University of Michigan Ann Arbor MI USA; ^3^ Division of Hematology/Oncology Department of Internal Medicine University of Michigan Ann Arbor MI USA; ^4^ Department of Internal Medicine University of Michigan Ann Arbor MI USA; ^5^ Department of Biostatistics School of Public Health University of Michigan Ann Arbor MI USA; ^6^ Department of Radiation Oncology and Molecular Radiation Sciences Johns Hopkins University Baltimore MD USA; ^7^ Department of Urology University of Michigan Ann Arbor MI USA; ^8^ Hunter Holmes McGuire Veterans Affairs Healthcare System Department of Radiation Oncology Virginia Commonwealth University Richmond VA USA; ^9^ Center for Innovation to Implementation Veterans Affairs Palo Alto Health Care System and the Department of Urology Stanford University Stanford CA USA; ^10^ Department of Learning Health Sciences University of Michigan Ann Arbor MI USA

**Keywords:** mass screening, prostatectomy, prostate‐specific antigen, prostatic neoplasms, survivorship

## Abstract

**Background:**

Although prostate‐specific antigen (PSA) testing is used for prostate cancer detection and posttreatment surveillance, thresholds in these settings differ. The screening cutoff of 4.0 ng/mL may be inappropriately used during postsurgery surveillance, where 0.2 ng/mL is typically used, creating missed opportunities for effective salvage radiation treatment. We performed a study to determine whether guideline concordance with annual postoperative PSA surveillance increases when PSA values exceed 4 ng/mL, which represents a screening threshold that is not relevant after surgery.

**Methods:**

We used US Veterans Health Administration data to perform a retrospective longitudinal cohort study of men diagnosed with nonmetastatic prostate cancer from 2005 to 2008 who underwent radical prostatectomy. We used logistic regression to examine the association between postoperative PSA levels and receipt of an annual PSA test.

**Results:**

Among 10 400 men and 38 901 person‐years of follow‐up, annual guideline concordance decreased from 95% in year 1 to 79% in year 7. After adjustment, guideline concordance was lower for the youngest and oldest men, Black, and unmarried men. Guideline concordance significantly increased as PSA exceeded 4 ng/mL (adjusted odds ratio 2.20 PSA > 4‐6 ng/mL vs PSA > 1‐4 ng/mL, 95% confidence interval 1.20‐4.03; *P* = .01).

**Conclusions:**

Guideline concordance with prostate cancer surveillance increased when PSA values exceeded 4 ng/mL, suggesting a screening threshold not relevant after prostate cancer surgery, where 0.2 ng/mL is considered treatment failure, is impacting cancer surveillance quality. Clarification of PSA thresholds for early detection vs cancer surveillance, as well as emphasizing adherence for younger and Black men, appears warranted.

## Graphical abstract

Evidence suggests that prostate‐specific antigen screening thresholds might be inappropriately applied in the postsurgical surveillance setting. Future work should emphasize strategies to clarify these two different thresholds in order to prevent missed opportunities for early postsurgery salvage therapy.

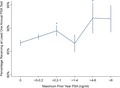

## 1 INTRODUCTION

After radical prostatectomy, up to one‐third of men will experience a rising prostate‐specific antigen (PSA) level indicating recurrent or persistent prostate cancer.^1^, ^2^ Given evidence supporting early treatment of recurrent disease (eg, at PSA < 0.5 ng/mL),^3^, ^4^, ^5^, ^6^, ^7^ guidelines recommend annual PSA surveillance for men treated surgically for prostate cancer.^8^

However, determinants of guideline adherence and quality of cancer surveillance after prostate cancer surgery remain unclear. Medicare data for men over 65 years of age suggest annual adherence is initially high, but declines over time.^9^, ^10^ Surveillance among younger and Black men, who potentially have the most to gain from appropriate cancer surveillance and early salvage treatment due to greater life expectancy^11^ and higher risk disease,^12^, ^13^ has not been well‐described. Lastly, prior studies have not examined whether postoperative PSA values influence surveillance patterns. We wondered whether providers might be mistakenly using common screening cutoffs (eg, 4 ng/mL) as their primary threshold for doing more or less aggressive cancer surveillance in the post‐prostatectomy setting. The screening cutoffs are not appropriate after prostate cancer surgery, where much lower cutoffs (ie, 0.2 ng/mL) indicate treatment failure and prompt salvage radiotherapy.^14^ This raises concerns about missed opportunities for potentially life‐saving salvage radiation treatment.^4^ With over 3 million US prostate cancer survivors,^15^ clarity regarding quality of cancer surveillance and areas for improvement is needed.

For these reasons, we conducted a study to better understand guideline concordance and quality of PSA surveillance among men undergoing prostate cancer surgery in a national integrated delivery system. We used Veterans Health Administration (VA) data for this study, which, in contrast to Medicare data, have a relatively large population of younger and Black men, as well as PSA test values that drive decision‐making. The VA is generally found to perform similarly or better than Medicare in non‐VA hospitals on reported quality measures and outcomes, including those for cancer.^16^, ^17^, ^18^ Our findings identify broad opportunities for survivorship care planning efforts to improve the quality of prostate cancer surveillance and care.

## 2 METHODS

### 2.1 Study population

Our study population included men with biopsy‐proven incident prostate cancer diagnosed between January 2005 and December 2008, with follow‐up through 2012, identified using the Veterans Administration Central Cancer Registry. We included men with nonmetastatic prostate cancer who underwent radical prostatectomy within 1 year of diagnosis. We excluded men with a prior diagnosis of prostate or other malignancy, hospice enrollment within 30 days, diagnosis at autopsy, and androgen deprivation therapy (ADT) or radiotherapy prior to or within 1 year of surgery. We also excluded men who died within 2 years following radical prostatectomy to allow for at least two annual PSA tests for each patient. We extracted cancer registry data, including biopsy Gleason score, pretreatment PSA level, clinical tumor stage, and surgical margin status. We collected demographic information, including age at diagnosis, race, ethnicity, marital status, and ZIP code. We used diagnosis codes to calculate comorbidity scores.^19^

### 2.2 PSA surveillance after surgery

We used laboratory files within the VA Corporate Data Warehouse to identify the date and value of PSA tests after surgery. We started the surveillance period 60 days after surgery through 31 December 2012 and defined guideline concordance as receiving at least one PSA test within each 12‐month period. Each patient was eligible for at least 4 years of follow‐up. Next, we determined the maximum PSA value in the preceding year for each person‐year of follow‐up.

Lastly, we used administrative and pharmacy claims data to identify salvage radiotherapy or ADT after surgery. We censored patients at receipt of these treatments since our primary objective for this study was to understand surveillance patterns and quality after surgery, but before treatment for recurrence. Patients being treated for recurrence typically follow with medical or radiation oncologists, rather than primary care providers as might happen after surgery. Given the risk of ascertainment bias due to patients receiving salvage treatment outside of the VA, we also performed a sensitivity analysis in which patients were not censored in this fashion. We also censored patients at death.

### 2.3 Guideline concordance and quality of PSA surveillance

Our primary outcome was receipt of annual guideline‐concordant PSA surveillance over the study period described above.

### 2.4 Assessing the association between PSA values and guideline concordance after surgery

We hypothesized that prior postoperative PSA values would influence guideline concordance. More specifically, we hypothesized that some providers performing surveillance might confuse the laboratory reference thresholds for screening (ie, 4 ng/mL) and biochemical failure after surgery (>0.2 ng/mL), and would be more likely to refer patients for salvage treatment only when their PSA values surpassed 4 ng/mL, resulting in increased PSA surveillance above this threshold and lower quality care. Additionally, we hypothesized that an undetectable (vs detectable) PSA level in the prior year would be associated with less concern for recurrence and subsequently decreased surveillance, and vice‐versa.

Our primary exposure was therefore the prior year's postoperative PSA value. We defined several categories based on thresholds corresponding to existing guidelines, including 0.2 ng/mL as a common definition of biochemical failure,^20^, ^21^ and 4 ng/mL as the upper limit of normal in most laboratories.^22^ In order to help differentiate a threshold effect at 4 ng/mL vs a continuous increase, we defined two categories for PSA values greater than 4 ng/mL: >4‐6 and >6 ng/mL. This resulted in the following categories for the prior year's PSA value for each patient: 0, >0‐0.2, >0.2‐1, >1‐4, >4‐6, and >6 ng/mL. If no PSA was obtained in a given year, we used the last value carried forward approach. If no PSA values were obtained in any of the preceding years, the prior year's PSA value was defined as missing. The primary hypothesis was that PSA concordance would be greater for prior year postoperative PSA values of >4‐6 vs >1‐4.

### 2.5 Statistical analysis

We used descriptive statistics to characterize our cohort. Less than 5% of baseline demographic variables were missing for all categories, except for clinical tumor stage (6.0% missing) and surgical margins (14% missing). We therefore performed multiple imputation, generating 10 imputations using all baseline clinical and demographic variables, year of diagnosis, region (geographically divided administrative areas called Veterans Integrated Services Networks), and year 1 PSA guideline concordance.

Next, we calculated the annual guideline concordance rate for each year of follow‐up overall, and by race/ethnicity. We examined bivariable relationships between guideline concordance and the preceding year's maximum PSA values, as well as baseline clinical and demographic factors, and year of diagnosis, using multilevel logistic regression modeling to account for clustering at the patient level.^23^ To further explain the relationship between postoperative PSA values and guideline concordance, we generated a multilevel, multivariable model to account for both patient‐level clustering and baseline characteristics.

Analyses were performed using Stata Version 15. We considered a two‐sided *P* value of <.05 as statistically significant. This study was approved by the VA Ann Arbor Healthcare System Institutional Review Board and a waiver of informed consent was received.

## 3 RESULTS

As shown in Table 1, we evaluated annual PSA surveillance for 10 400 men treated with radical prostatectomy. There was a broad range of ages from 34 to 95 years, with a mean of 62 years. Nearly one‐quarter of prostate cancer survivors were Black (23%), 57% were married, 55% had a comorbidity score of 0, and 19% lived in a rural area. Most men had intermediate risk disease and negative surgical margins. Receipt of postoperative ADT and radiotherapy was uncommon, with less than one in 10 men receiving these treatments in our delivery system.

**Table 1:** Characteristics of 10 400 men treated with radical prostatectomy. Non‐imputed and mean of 10 imputed datasets are shown

Characteristic	Non‐imputed	Imputed
	(N = 10 400)	(N = 10 400)
Age at diagnosis		
Mean (SD)	61.6 (6.8)	61.6 (6.8)
Race (no, %)		
White	7695 (74.4%)	7899 (76.0%)
Black	2352 (23.2%)	2392 (23.0%)
Other	106 (1.0%)	109 (1.0%)
Missing	247 (2.4%)	0 (0.0%)
Ethnicity (no, %)		
Not Hispanic	9805 (94.2%)	9874 (94.9%)
Hispanic	523 (5.1%)	527 (5.1%)
Missing	72 (0.7%)	0 (0.0%)
Married (no, %)		
Not married	4431 (42.6%)	4433 (42.6%)
Married	5964 (57.4%)	5966 (57.4%)
Missing	5 (0.0%)	0 (0.0%)
Rural (no, %)		
0	8397 (80.7%)	8401 (80.8%)
1	1997 (19.2%)	1999 (19.2%)
Missing	6 (0.1%)	0 (0.0%)
Charlson score (no, %)		
0	5694 (55.4%)	5759 (55.4%)
1	2590 (25.2%)	2634 (25.3%)
≥2	1987 (19.3%)	2008 (19.3%)
Missing	129 (1.3%)	0 (0.0%)
Gleason score (no, %)		
6	4511 (43.5%)	4522 (43.5%)
7	4963 (47.8%)	4976 (47.8%)
8‐10	901 (8.7%)	903 (8.7%)
Missing	25 (0.2%)	0 (0.0%)
PSA (ng/mL) (no, %)		
≤10	8885 (86.4%)	8991 (86.5%)
>10 and ≤20	1113 (10.8%)	1125 (10.8%)
>20	280 (2.7%)	284 (2.7%)
Missing	122 (1.2%)	0 (0.0%)
Clinical T stage (no, %)		
T1c‐T2a	8392 (85.5%)	8898 (85.6%)
T2b	342 (3.5%)	361 (3.5%)
≥T2c	1078 (11.0%)	1141 (11.0%)
Missing	588 (6.0%)	0 (0.0%)
Surgical margins (no, %)		
Negative	7258 (80.7%)	8431 (81.1%)
Positive	1734 (19.3%)	1969 (18.9%)
Missing	1408 (15.7%)	0 (0.0%)
Had ADT after surgery	867 (8.3%)	867 (8.3%)
Had RT after surgery	603 (5.8%)	603 (5.8%)
Time from surgery to postop ADT (y)		
Mean (SD)	3.8 (1.9)	3.8 (1.9)
Time from surgery to first RT (y)		
Mean (SD)	3.18 (1.7)	3.2 (1.7)

Abbreviations: ADT, androgen deprivation therapy; PSA, prostate‐specific antigen.

**Figure 1:**
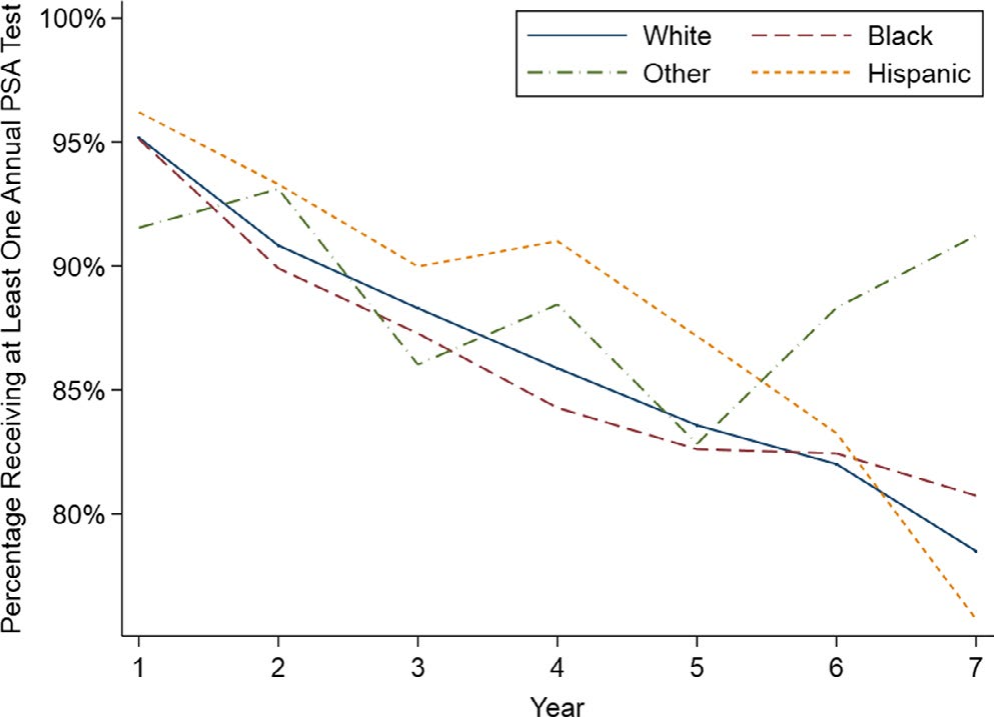
Annual guideline concordance for prostate‐specific antigen (PSA) surveillance among 10 400 patients after radical prostatectomy in a national delivery system according to race and ethnicity

Annual guideline concordance was initially very high (95% in year 1), but declined over time (79% in year 7), with Figure 1 demonstrating rates by race/ethnicity. The average patient‐level guideline concordance was 87%. On unadjusted bivariate analyses, annual guideline concordance was less common among patients who were not married, and more common among patients with Gleason 7 disease (vs 6), positive margins, and Hispanic ethnicity (*P* < .05). We also found an age‐related association with guideline concordance for both age and age^2^, indicating that patients at the extremes of age (ie, youngest and oldest) had lower guideline concordance than those of intermediate ages (*P* < .05). Guideline concordance significantly increased when prior year PSA values exceeded 4 ng/mL (odds ratio [OR] 1.94 PSA > 4‐6 ng/mL vs PSA > 1‐4 ng/mL, 95% confidence interval [CI] 1.07‐3.50; *P* = .03).

**Figure 2:**
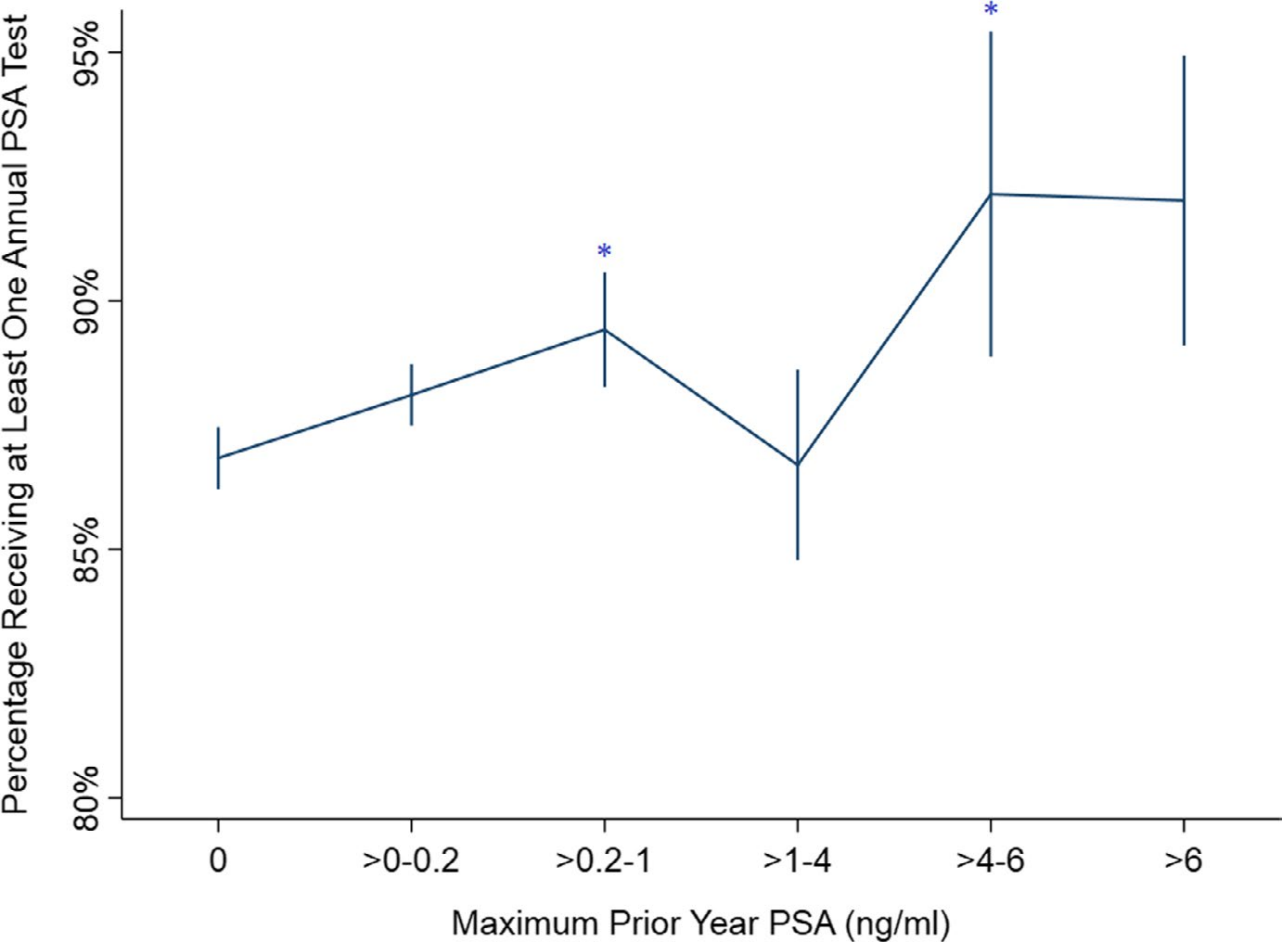
Guideline concordance by prior year prostate‐specific antigen (PSA) values. Model‐predicted annual PSA guideline concordance by prior year PSA value among 10 400 patients surviving at least 2 years without receipt of salvage therapy. *denotes statistically significant guideline concordance compared to PSA > 1 and ≤4 (*P* < .05)

On multivariable analysis, Black race (adjusted OR [aOR] 0.86, 95% CI 0.95‐0.99; *P* = .03) and initial PSA levels between 10 and 20 ng/mL (Table 2) became significant predictors of lower guideline concordance, and Gleason 7 disease was no longer significant. Age, age^2^, Hispanic ethnicity, and marital status remained significant predictors (*P* < .01), as did the prior year's PSA values, demonstrating a threshold effect illustrated in Figure 2 when PSA values exceeded a threshold of 4 ng/mL (marginal prediction, averaged over all other variables in the multivariable model, aOR 2.19 for PSA > 4‐6 ng/mL vs PSA >1‐4 ng/mL, 95% CI 1.20‐4.03; *P* = .01; Table 2).

**Table 2:** Multilevel, multivariable model. Adjusted odds of guideline‐concordant prostate cancer surveillance with annual PSA testing among 10 400 men treated with radical prostatectomy using multilevel, multivariable logistic regression (clustering at patient level)

Characteristic	Level	Adjusted odds ratio	95% CI		*P* value
Age at diagnosis		1.59	1.47	1.73	<.001
Age at diagnosis^2^		1.00	1.00	1.00	<.001
Race	White	1.00	.	.	.
	Black	0.86	0.75	0.99	.03
	Other	1.19	0.69	2.06	.54
Ethnicity	Not Hispanic	1.00	.	.	.
	Hispanic	1.41	1.09	1.83	.01
Marital status	Not married	1.00	.	.	.
	Married	1.21	1.09	1.35	<.001
Rurality	Rural	1.09	0.95	1.26	.20
Charlson score	0	1.00	.	.	.
	1	1.07	0.94	1.22	.31
	≥2	0.99	0.86	1.14	.86
Gleason score	6	1.00	.	.	.
	7	1.10	0.98	1.23	.10
	8‐10	1.08	0.88	1.32	.48
Baseline PSA	≤10	1.00	.	.	.
	>10 and ≤20	0.82	0.69	0.97	.02
	>20	0.89	0.64	1.25	.50
Clinical T stage	T1c‐T2a	1.00	.	.	.
	T2b	0.87	0.65	1.17	.35
	≥T2c	0.94	0.79	1.12	.50
Surgical margins	Negative	1.00	.	.	.
	Positive	1.17	1.01	1.35	.04
Year posttreatment	2	1.00	.	.	.
	3	0.61	0.54	0.68	<.001
	4	0.43	0.38	0.48	<.001
	5	0.33	0.29	0.37	<.001
	6	0.28	0.24	0.32	<.001
	7	0.20	0.17	0.25	<.001
Prior year maximum PSA level (ng/mL)	0	1.02	0.80	1.30	.89
	>0‐0.2	1.19	0.93	1.52	.16
	>0.2‐1	1.42	1.09	1.86	.01
	>1‐4	1.00	.	.	.
	>4‐6	2.20	1.20	4.03	.01
	>6	2.15	1.25	3.70	.01
	No prior postop PSA	0.11	0.08	0.15	<.001
Constant		0.00	0.00	0.00	<.001
lnsig2u		2.84	2.60	3.12	<.001

Abbreviation: PSA, prostate‐specific antigen.

**Figure 3:**
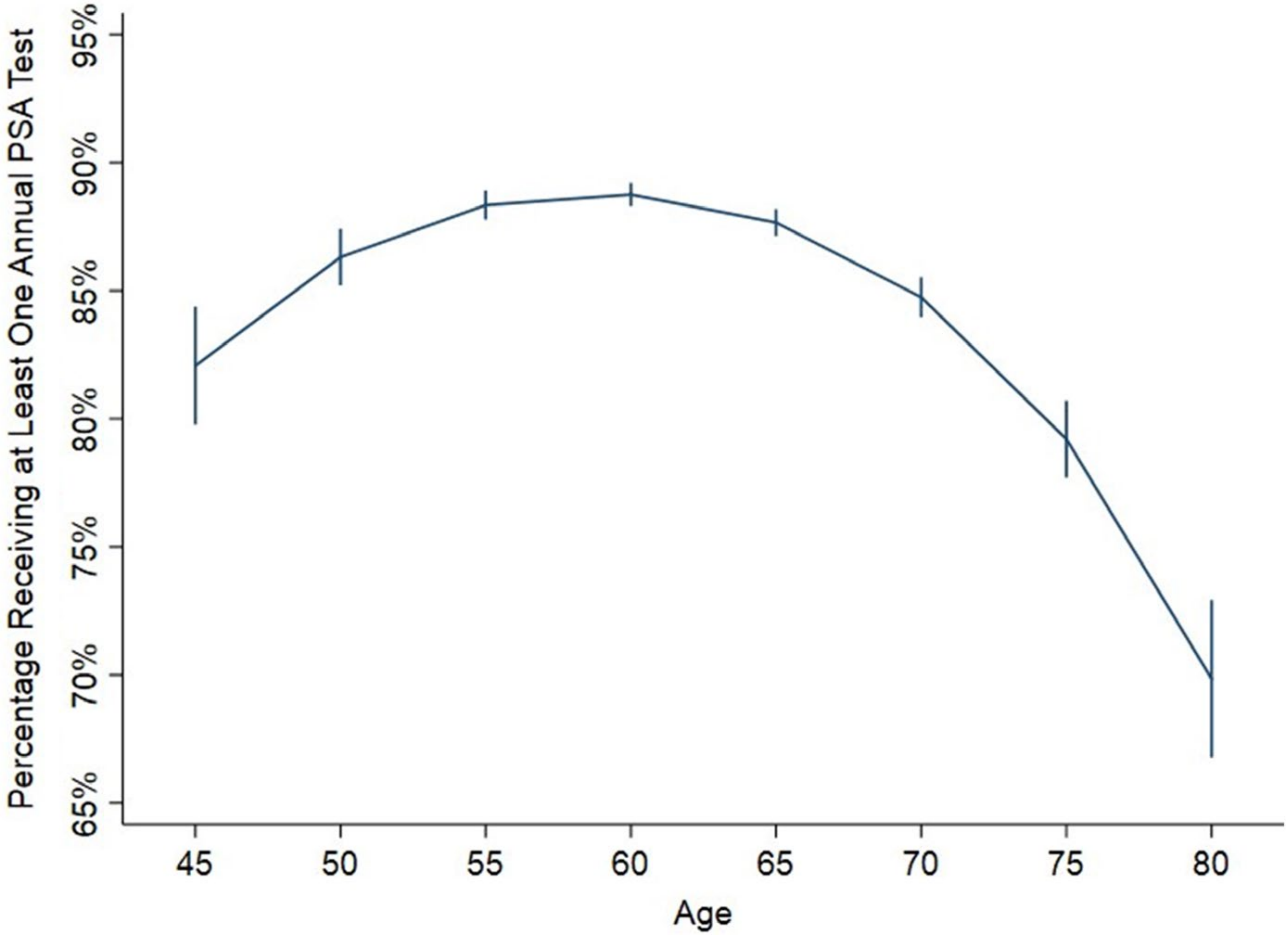
Model‐predicted annual prostate‐specific antigen (PSA) guideline concordance after radical prostatectomy by age

Exploratory analysis for other PSA values revealed that concordance sequentially increased for PSA values of 0, >0‐0.2, and >0.2‐1 ng/mL, but subsequently decreased for values of >1‐4 (aOR 1.42 for >0.2‐1 ng/mL vs 1.00 for >1‐4 ng/mL, 95% CI 1.09‐1.86; *P* = .009; Table 2). Figure 3 illustrates worse annual adjusted surveillance at the extremes of age (marginal prediction, averaged over all other variables in the model).

In our sensitivity analysis, in which patients were not censored at the time of receipt of radiotherapy or ADT, significance was unchanged for all variables except Gleason 7 disease, which became significant (vs Gleason 6, aOR 1.13; *P* = .03; 95% CI 1.01‐1.27) and baseline PSA, which was no longer significant. A prior year PSA of >4‐6 ng/mL continued to predict for increased surveillance compared to PSA > 1‐4 ng/mL (aOR 2.34; 95% CI 1.30‐4.21; *P* = .005).

## 4 DISCUSSION

This national study examined guideline concordance with annual PSA surveillance after radical prostatectomy, including the unique relationship between postoperative PSA values and quality of cancer surveillance. We found guideline concordance sharply increases when the preceding year's PSA values rose beyond 4 ng/mL. This suggests that providers may be using a threshold not relevant in the postoperative setting, but traditionally associated with early detection (ie, screening) as a stimulus to intensify cancer surveillance. In addition, the lower rates of surveillance we describe beneath the threshold likely lead to less effective salvage treatment. Although overall guideline concordance was high, we found lower guideline concordance among men who were Black, not married, and at the extremes of age including younger men in the full model.

Our most notable finding is the association between guideline concordance and the preceding year's PSA values. PSA values might influence cancer surveillance patterns for a number of reasons. First, low or undetectable values might provide premature reassurance to patients and providers, decreasing subsequent surveillance. Indeed, we observed that having an undetectable PSA was associated with a lower likelihood of surveillance than having PSA levels between 0.2 and 1. However, patients with initially undetectable PSA levels remain at risk of prostate cancer recurrence and local disease recurrence for up to 10 or more years after surgery.^1^, ^21^

A second explanation for the association between PSA values and surveillance patterns likely involves transitions of care from primary care providers to urologists and oncologists in the setting of recurrence (ie, higher PSA values, particularly over 4 ng/mL). When patients experience biochemical recurrence, providers may refer them back to urology, or medical or radiation oncology for salvage treatment. These referrals likely result in more frequent PSA monitoring with the goals of establishing the PSA trajectory before deciding upon further treatment or closely monitoring PSA levels after radiotherapy or ADT initiation. Indeed, quality prostate cancer surveillance after surgery should lead to earlier salvage treatment (ie, at PSA levels <0.5 ng/mL) with subsequent better progression‐free, metastasis‐free, and overall survival outcomes as demonstrated in randomized trials.^3^, ^4^, ^7^

Interestingly, we identified a striking increase in guideline concordance once the prior year PSA values crossed the threshold of 4 ng/mL. The lack of evidence of any additional increase at other higher PSA thresholds (eg, 6 ng/mL) further suggests that 4 ng/mL represents a true threshold for increased surveillance, as opposed to an arbitrary marker of increased disease burden leading to more aggressive PSA monitoring. As described above, this cutoff should not be applied after prostatectomy where the definition of biochemical recurrence remains much lower (eg, detectable or ≥0.1‐0.2 ng/mL).^8^, ^20^ This is common knowledge for oncologists treating prostate cancer (ie, urologists, radiation, and medical oncologists), but may not be appropriately conveyed to primary care providers during survivorship care transitions, or crowded out of the PCPs operational memory by the more common exposure to and use of screening thresholds for prostate cancer.^24^ Both primary care and oncology providers may relate to our findings given that the PSA reference ranges embedded in laboratory reports are based on screening thresholds.

Regardless of the cause, decreased rates of surveillance will increase the risk of delayed referrals and missed opportunities for effective salvage therapy. Based on our findings, one way to increase the quality of survivorship care would be to try to find ways to disseminate knowledge about PSA thresholds for disease recurrence. As cancer survivorship care plans are being implemented nationally within and outside VA,^25^ future efforts should examine whether these plans lead to increases in guideline concordance and quality of cancer surveillance. Another relatively easy intervention would be for the laboratory to provide separate PSA test reference ranges for screening and surveillance after prostate cancer surgery, prompting referral to oncologists for values ≥0.2 ng/mL.

Current surveillance PSA guidelines apply to all patients treated for prostate cancer, regardless of recurrence risk. Despite this, we observed differences on the basis of clinical characteristics. We found surveillance was higher among patients with positive margins. Although these patients are more likely to experience recurrence,^26^ they are also more likely than patients with negative margins to be successfully salvaged by radiation in the setting of biochemical failure,^5^ highlighting the importance of surveillance in this population. We did not observe differences according to comorbidity, though did identify decreasing surveillance among the oldest patients consistent with previous studies.^9^, ^10^ Until better science emerges to help tailor PSA surveillance based on disease risk^27^ and life expectancy,^28^ it remains a guideline‐recommended low‐cost, low‐risk intervention for men of all risk groups whose disease warranted definitive treatment with surgery.

Another novel and important finding in our study was lower guideline concordance among the youngest surgical patients. This finding from a national delivery system differs from previous reports of younger patients in smaller cohorts.^9^ Decreased surveillance among younger patients raises concerns because they have a higher risk of dying of prostate cancer when they present with recurrence, often with aggressive disease compared to older men.^29^ Moreover, due to lower competing mortality risks,^11^ they also have a higher risk of dying from prostate cancer if salvage opportunities are missed due to poor quality surveillance (ie, inappropriate PSA thresholds).

We also demonstrated a disparity on the basis of race, with lower surveillance for Black patients similar to prior studies.^9^, ^10^ This disparity is particularly concerning, given Black men experience higher disease incidence and mortality.^30^ Fortunately, the magnitude of this difference was small. Hispanic men actually had higher guideline concordance in this study than other populations, contrary to what has been demonstrated in the Medicare population.^10^ Additionally, unmarried patients fare worse with respect to many health and prostate cancer‐specific outcomes^31^ including cancer surveillance in this study, suggesting that this population might also benefit from tailored interventions to ensure quality prostate cancer surveillance.

Overall, guideline concordance rates in our study were high and comparable to that of the Medicare population.^10^ This is notable because it demonstrates that the VA is able to provide high‐quality care for veterans,^16^, ^32^ despite the fact that the population has higher prevalence of characteristics often associated with lower quality care, including low income, racial/ethnic minority status, and being unmarried. Future studies should emphasize the ways in which the VA succeeds at providing high‐quality care to traditionally underserved populations (eg, telehealth, transportation support), as these principles could be applied in other settings.

Because our study was performed within the integrated Veterans Administration Healthcare system, it may not completely generalize the overall population or patients who receive more fragmented prostate cancer survivorship care. Nonetheless, our findings raise concerns about quality of surveillance that likely apply to non‐VA settings. Another limitation is the risk of ascertainment bias due to patients undergoing PSA testing or receiving primary care provider (ADT) or radiotherapy (RT) outside of the VA. However, our sensitivity analysis in which patients were not censored based on ADT/RT receipt demonstrated a minimal impact on the significance of our findings. Our approach differed from that of other studies in which salvage and palliative therapies were not ascertained, obscuring other factors associated with surveillance.^9^, ^10^ By excluding PSA values obtained once patients established care with oncologists to obtain ADT or radiotherapy, our approach has the advantage of quantifying surveillance rates that more closely reflect the period for which surveillance was designed. With respect to PSA under ascertainment, patients would only have better guideline concordance if PSA tests were obtained elsewhere and there is no reason to suspect that this would have affected the relationships between PSA values or greater and less than 4 ng/mL.

## 5 CONCLUSIONS

In conclusion, while the majority of patients received guideline‐concordant PSA surveillance after prostate cancer surgery in this national delivery system, we found guideline concordance sharply increased when PSA values exceeded 4 ng/mL, suggesting providers may be using a screening threshold to make decisions about cancer surveillance. The screening threshold of 4 ng/mL is not relevant in the post‐prostatectomy setting where 0.2 ng/mL is considered treatment failure, warranting salvage treatment discussions. Until further research demonstrates the clinical utility and feasibility of tailored cancer surveillance strategies, efforts should be made to increase surveillance for all patients whose cancer warrants treatment, including those with high‐risk disease and barriers to care. New laboratory reference ranges may clarify PSA thresholds for early detection vs cancer surveillance, and survivorship care plans should emphasize adherence for younger and Black men to avoid missed opportunities for salvage treatment and improve quality of care.

## CONFLICT OF INTEREST

None declared.

## AUTHOR CONTRIBUTIONS

Christina Hunter Chapman: conceptualization, data curation, formal analysis, project administration, investigation, methodology, supervision, validation, visualization, writing—original draft, and writing—review and editing. Megan EV Caram: conceptualization, methodology, writing—original draft, and writing—review and editing. Archana Radhakrisnan: conceptualization and writing—review and editing. Alexander Tsodikov: methodology, writing—review and editing. Curtiland Deville: conceptualization, methodology, writing—review and editing. Jennifer Burns: data curation, formal analysis, investigation, methodology, project administration, writing—review and editing. Alexander Zazlavsky: methodology, writing—review and editing. Michael Chang: methodology, writing—review and editing. John T Leppert: methodology, writing—review and editing. Timothy Hofer: methodology, writing—review and editing. Anne E. Sales: conceptualization, methodology, project administration, supervision, writing—review and editing. Sarah T. Hawley: conceptualization, methodology, supervision, and writing—review and editing. Brent K. Hollenbeck: conceptualization, methodology, supervision, and writing—review and editing. Ted A. Skolarus: conceptualization, data curation, formal analysis, funding acquisition, investigation, methodology, project administration, resources, software, supervision, validation, visualization, writing—original draft, and writing—review and editing. Precis for use in Table of Contents: Evidence suggests that PSA screening thresholds might be inappropriately applied in the postsurgical surveillance setting. Future work should emphasize strategies to clarify these two different thresholds in order to prevent missed opportunities for early postsurgery salvage therapy.

## Data Availability

Data are not shared.
