# Supplementary material for: Association between PSA values and surveillance quality after prostate cancer surgery
Source: Cancer Med. 2019 Nov 5;8(18):7903–12. doi: 10.1002/cam4.2663 (PMC6912050; doi:10.1002/cam4.2663)
Supplement: Supplementary file 1 [file CAM4-8-7903-s001.doc]

**Figure S1**: Flow diagram demonstrates sample counts for included and excluded observations. Abbreviations: ADT, androgen deprivation therapy; RT, radiotherapy

Men with non-metastatic prostate cancer diagnosed between 1/1/2005 and 12/31/2008

n = 52,311

No surgery within one year of diagnosis

n=40,041

Surgery within one year of diagnosis
n=12,270

No PSA data available in laboratory file

n=56

At least one PSA value in laboratory file

N=12224

Surgery on or before 12/31/2009
n = 12208

Treated after 12/31/2009

n=16

Survived at least 2 years post-surgery
n = 11640

No ADT before surgery
n = 11206

Died in year 1 or 2

n=568

Received ADT before surgery

n=434

Received ADT or RT within one year after surgery
n = 10400

Received ADT or RT within one year after surgery n=806
